# Crosslink between Temozolomide and PD-L1 immune-checkpoint inhibition in glioblastoma multiforme

**DOI:** 10.1186/s12885-019-5308-y

**Published:** 2019-02-01

**Authors:** Sabrina Heynckes, Karam Daka, Pamela Franco, Annette Gaebelein, Jan Hendrik Frenking, Roberto Doria-Medina, Irina Mader, Daniel Delev, Oliver Schnell, Dieter Henrik Heiland

**Affiliations:** 1 Department of Neurosurgery, Medical Center University of Freiburg, Breisacher Straße 64, 79106 Freiburg, Germany; 2 Department of Neuroradiology, Medical Center - University of Freiburg, Freiburg, Germany; 3 Clinic for Neuropediatrics and Neurorehabilitation, Epilepsy Center for Children and Adolescents, Schön Klinik, Vogtareuth, Germany; 4 Department of Neurosurgery, University of Aachen, Aachen, Germany; 5 Faculty of Medicine, University of Freiburg, Freiburg, Germany

**Keywords:** Temozolomide, Recurrent GBM, PD-L1 immunotherapy

## Abstract

**Background:**

In recent years, PD-1/PD-L1 immune checkpoint inhibitors have improved cancer therapy in many tumor types, but no benefit of immune checkpoint therapy has been found in glioblastoma multiforme (GBM). Based on the results of our earlier work, which showed a reduction of PD-L1 expression in patients treated with temozolomide (TMZ), we aimed to investigate the link between TMZ therapy and the immune control point target PD-L1.

**Methods:**

RNA-sequencing data from de-novo and recurrent glioblastoma were analyzed by AutoPipe algorithm. Results were confirmed either in a cell model by two primary and one established GBM cell line and specimens of de-novo and recurrent GBM. PD-L1 and pathway activation of the JAK/STAT pathway was analyzed by quantitative real-time PCR and western blot.

**Results:**

We found a significant downregulation of the JAK/STAT pathway and immune response in recurrent tumors. The cell model showed an upregulation of PD-L1 after IFNγ treatment, while additional TMZ treatment lead to a reduction of PD-L1 expression and JAK/STAT pathway activation. These findings were confirmed in specimens of de-novo and recurrent glioblastoma.

**Conclusions:**

Our results suggest that TMZ therapy leads to a down-regulation of PD-L1 in primary GBM cells. These results support the clinical findings where PD-L1 is significantly reduced in recurrent GBMs. If the target is diminished, it may also lead to impaired efficacy of PD-1/PD-L1 inhibitors such as nivolumab.

## Background

Glioblastoma Multiforme (GBM) is the most common and malignant primary brain tumor in adults, with an annual incidence of 3–4 cases per 100,000 people in Europe [1, 2] and the United states [3]. In spite of the best available treatment, the prognosis for patients with GBM is poor, with a median survival of 14–16 months [4–8]. The standard-of-care treatment combines adjuvant radio- plus TMZ chemotherapy [9], however, the median increase of survival is limited [9]. In the last years, a new class of therapeutic drugs has revolutionized cancer therapy [10]. PD-1/PD-L1 immune checkpoint inhibitors such as pembrolizumab and nivolumab revealed a strong response and improvement in overall survival in various tumor entities such as metastatic melanoma [11], non-small-cell lung cancer (NSCLC) and head and neck tumors [12]. PD-L1 receptors on the surface of tumor cells interact with PD-1 positive myeloid cells and reduce immune-mediated cell damage [13]. In 2014, a large randomized phase III trial, Checkmate 143 (NCT02617589), was performed to explore the PD-1 inhibitor Nivolumab in patients with recurrent GBM tumors [14]. Despite high expectations, early results of the Checkmate 143 study surprisingly revealed no significant benefit of Nivolumab treatment for patients with a recurrent GBM [15]. In a recent study performed by our group, we analyzed *PD-L1* expression in de-novo and recurrent GBM samples [16]. Contrary to primary assumptions, we found a downregulation of *PD-L1* in recurrent GBM. Further, we identified extended TMZ therapy as significantly inverse correlated with *PD-L1* expression. This led us to further investigate the role of TMZ in PD-L1 regulation, which has so far been associated with various signaling pathways, in particular the activation of the interferon-gamma (IFN) pathway [17–19]. IFNγ is released by immune cells after activation of the immune system and partially controls immune response [20]. JAK/STAT pathway activation via the IFNγ receptor on the surface of the tumor cell leads to an increased expression of *Interferon-stimulated genes* (*ISGs*), including *PD-L1* [20]. Under physiological conditions, this mechanism contributes to immune homeostasis and limits inflammation [21].

The purpose of this study was to investigate the effect of TMZ on intracellular signaling with a special focus on the PD-L1 pathway. Therefrom we aimed to investigate potential synergistic or antagonistic effects that might result from combined treatment with TMZ and PD-1/PD-L1 inhibition.

## Methods

### Contact for reagent and resource sharing

Further information and requests for resources, raw data and reagents should be directed and will be fulfilled by the Contact: D. H. Heiland, dieter.henrik.heiland@uniklinik-freiburg.de.

### Ethical approval

For this study all included patients were diagnosed with a primary glioblastoma multiforme WHO grade IV (without known lower-grade lesion in the patient’s history), who underwent surgery at the Department of Neurosurgery of the Medical Center, University of Freiburg. The local ethics committee of the University of Freiburg approved data evaluation and experimental design (protocol 100,020/09 and 5565/15). The methods were carried out in accordance with the approved guidelines. Written informed consent was obtained.

### Cell culture

Brain tumor tissue was obtained during the neurosurgical tumor resection and further processed in sterile conditions under a tissue culture hood. First, the tissue was fragmented to small pieces and resuspended in cell-dissolving solution. The tissue fragments were centrifuged at 1000 rpm for 5 min and subsequently resuspended with 5 ml ACK Lysing Buffer to remove blood cells. The cells were finally resuspended in medium and transferred into a tissue culture flask.

### Cell treatment and environmental simulation

Two patient-derived cell lines were each divided into 4 groups, which were seeded in different dishes: the first group (ctrl) received no treatment and functioned as control group. The second group (IFNγ) was treated with IFNγ (100 ng/μl) to achieve activation of immune response pathways. The third group (TMZ) was treated with Temozolomide in a concentration of 75 μM to simulate standard-of-care chemotherapeutic treatment. To the fourth group (IFNγ+TMZ), 75 μM TMZ was added plus IFNγ (100 ng/μl). Treatment medium was always prepared freshly using serum-free cell culture medium and was directly administered to the cells after splitting, counting and seeding. After a treatment of 48 h, cells were harvested and frozen in the − 80 °C fridge for later RNA and Protein extraction. The same treatment set up was used for immunofluorescence experiments. All cell culture experiments were performed three times in biological independence.

### Immunoblotting

Cells were lysed using Radio Immuno Precipitation Buffer (RIPA buffer) and protease inhibitor on ice. Afterwards, the lysate was centrifuged at 14.000 rpm for 30 min at 4 °C. The supernatant was used to measure the protein concentration by NanoDrop. Laemmli buffer was added to the samples and the concentration was equalized. The specific, primary antibody was dissolved in 5% BSA TBS-0.1%T buffer, added to the membrane and incubated under constant agitation at 4 °C overnight. Used primary antibodies were: Anti-PD-L1 (rabbit, conc. 1:500, Cell Signaling), Anti-STAT3 (rabbit, conc. 1:500, Santa Cruz), Anti-phospho-STAT3 (rabbit, conc. 1:500, Santa Cruz) and Anti-α-Tubulin (mouse, conc. 1:1000, Abcam). A digital imager ChemiDoc XRS detected the chemiluminescence emanation from the membrane by transforming the signal into a digital image.

### Quantitative real-time PCR

RNA was extracted by All Prep Kit (Qiagen, Venlo, Netherlands) according to the manufacturer’s instructions. RNA integrity was measured using the Agilent RNA Nano Assay Bioanalyser 2100 (http://www.home.agilent.com) according to the manufacturer’s instructions. Primers were produced by life technology (www.lifetechnologies.com). The qRT-PCR reaction was performed using the SYBR Green PCR Master Kit. The PCR reaction was run using the 7900HT Fast Real-Time PCR System with the standard SYBR green protocol. Average cDNA quantities relative to a standard amplified gene (Housekeeper Gene: *18S*) were calculated using R-statistics.

### Immunofluorescence

For immunostaining, cells were cultivated on slides and fixed with 3% formaldehyde for 10 min at room temperature. Immunostaining was performed as recently published by our group [22]. A Fluoview FV10i confocal microscope from Olympus was used for fluorescence microscopy. All measurements and image processing were performed using the company’s software. Optical magnification settings of 10X and 60X with oil were used. A laser with wavelengths of 647 nm (ALEXA 647), 594 nm (ALEXA 594) and 358 nm (DAPI) was selected for imaging. Laser power was manually optimized and used with equal settings for all imaged samples.

### Predictive analysis of transcriptome data

In order to identify differentially expressed genes between de-novo GBM and recurrent tumors, RNA sequencing data from Bao and colleagues [23] were downloaded from GEO containing 272 patients. The *AutoPipe R-package,* an algorithm based on a supervised-condition specific machine-learning approach (github.com/heilandd) was used to identify gene expression changes between de-novo and recurrent GBM samples. This algorithm calculated the optimal number of clusters in a first step. By using clinical features such as recurrence status, the algorithm predicts genes who are exclusively expressed in different groups by a machine-learning approach as described earlier [24]. In order to identify the core samples of each cluster, we sorted out patients of each cluster based on a negative silhouette width. The given prediction values of each cluster were further used to analyze functional aspects.

### Functional analysis by enrichment analysis

A permutation-based pre-ranked Gene Set Enrichment Analysis (GSEA) was applied to each module to verify its biological functions and pathways [25] by using the predefined gene sets of the Molecular Signature Database v5.1. Enrichment score was calculated by the rank order of gene/metabolite computed by random forest accuracy [25]. For significant enrichment, *p*-values were adjusted by FDR. Gene Set Variation Analysis (GSVA) was performed with the GSVA package implemented in R-software. The analysis was based on a non-parametric unsupervised approach, which transformed a classic gene matrix (gene-by-sample) into a gene set by sample matrix and resulted in an enrichment score for each sample and pathway [26].

## Results

### Recurrent glioblastoma are marked by a distinct transcriptomic profile

**Fig. 1:**
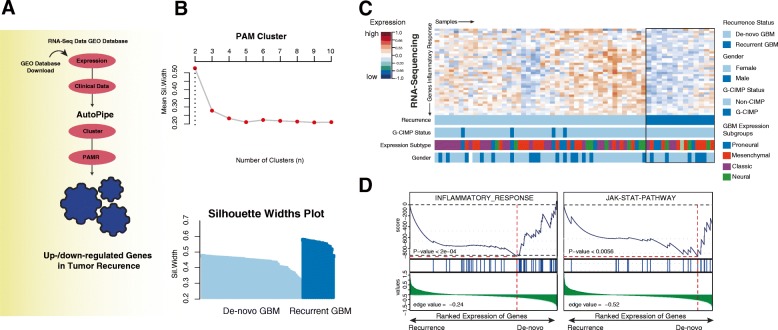
**a** Illustration of the workflow displaying the bioinformatical approach. **b** The upper panel shows a plot illustrating the optimal number of clusters. The optimal number of clusters was achieved by PAM clustering from 2 to 10 number of clusters by calculating the mean silhouette widths. In the bottom plot, the silhouette widths of both clusters are shown ordered by de-novo (light blue) and recurrent GBM (dark blue). **c** Heatmap of genes of the INFLAMMATORY RESPONSE (Hallmark genesets) based on the RNA-Sequencing data published by Bao and colleagues [23]. High and low gene expression levels are displayed in red and blue color, respectively. In the bottom panel of the heatmap, individual properties such as expression subgroup, CpG island methylation phenotype (CIMP) status and gender are shown. The explanation of each property is given on the right side. **d** Two plots displays a gene set enrichment analysis (GSEA) of pathways that were significantly downregulated in recurrent glioma

We started our examination by analyzing RNA-sequencing data of de-novo and recurrent GBM patients in order to identify transcriptomic patterns that occur predominantly in recurrent GBM (Fig. 1a). In a next step, we used RNA-seq data from Bao and colleagues [23] and performed a supervised clustering followed by gene set enrichment analysis (GSEA) to uncover pathway activation (Fig. 1b-c). In line with our previous results, the inflammatory response and JAK-STAT pathway was found to be significantly decreased, which has been described as the major driver for *PD-L1* expression. We hypothesized that differences in JAK/STAT pathway activation with consecutive downregulation of *PD-L1* in the recurrent state could be related to previous therapy such as radiotherapy or TMZ therapy. Even though there is a well-known immune sensitizing effect of radiotherapy, we focused on the role of TMZ and its potential immune suppressive effect on tumor cells.

### Temozolomid treatment decreases PD-L1 expression

**Fig. 2:**
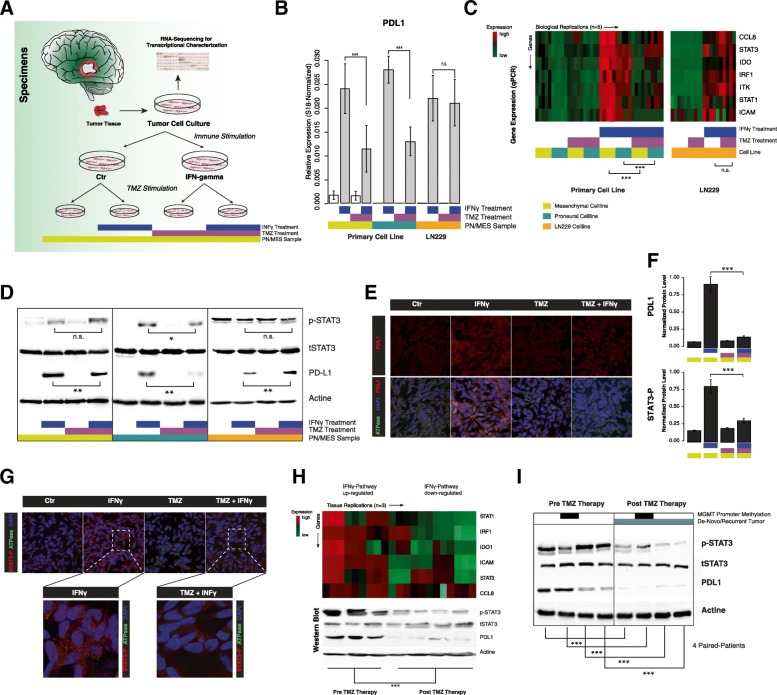
**a** Illustaration of the experimental workflow. **b** Bar plot of gene expression levels of *PD-L1* in three different cell-lines. Cell treatment condition and cell type is displayed at the bottom. **c** Gene expression heatmap of all experimental conditions with five biological replications and three cell lines. Arrows mark the direction of samples and genes. High and low gene expression levels of the JAK/STAT pathway panel is displayed in red and green color, respectively. Cell treatment condition and cell type is displayed at the bottom. **c** Immunoblot of all cell lines and experimental conditions. Actine was used as loading control. **d** Immunostaining of PD-L1 in GSC 168 cell lines (proneural). In the upper panel, PD-L1 is shown and a quantification is given on the right side (**f**). In the bottom panel, PD-L1 is merged with ATPase (marks cell membrane) and DAPI (marks nucleoli). **g** In the upper panel, STAT3 phosphorylation is displayed within each experimental condition. High resolution images of TMZ and TMZ plus IFNγ are shown in the bottom panel. A quantification of the STAT3 phosphorylation level is given in (**f**). **h** Gene expression heatmap of the JAK/STAT pathway in de-novo and recurrent GBM (upper plot) combined with the STAT3 phosphorylation and PD-L1 protein levels. High and low gene expression levels of the JAK/STAT pathway panel are displayed in red and green color, respectively. **i** Immunoblots of 4 patients with primary and recurrent GBM selected because of exceptionally high PD-L1 levels in de-novo tumor tissue. Significance level is displayed as following: * *p* < 0.05, ** *p* < 0.01, *** *p* < 0.001

Next, we aimed to analyze the effect of TMZ on *PD-L1* expression in three different cell lines. As previously shown by our group, *PD-L1* expression is distributed differently within all molecular subgroups [27]: especially tumors with a mesenchymal signature showed high levels of *PD-L1*. In order to respect subgroup properties, we used a commercial cell-line (LN229) and two primary glioma-stem-like cell lines (GSC168 and GSC-HE01JP), which were characterized as proneural or mesenchymal, respectively. An overview of the experimental workflow is given in Fig. 2a. First, we analyzed *PD-L1* expression in all three cell lines. In untreated control cell culture samples, *PD-L1* expression was only measurable in the primary mesenchymal cell line (GSC HE01JP), Fig. 2b. We hypothesized that the cell lines require an external stimulus that mimics the presence of the immune system in order to drive *PD-L1* expression. We administered a treatment with IFNγ as an immune stimulator to the cells, which increased the *PD-L1* expression significantly in all three cell lines (*p* < 0.01), Fig. 2b. In a next step, we analyzed the effects of additional TMZ treatment. In the primary cell lines GSC 168 and HE01JP, a significant loss of *PD-L1* upregulation was observed after a 48 h TMZ plus IFNγ treatment (*p* < 0.001). In contrast to the primary cell lines, commercial LN229 cells showed no significant differences of *PD-L1* expression after TMZ treatment, Fig. 2b.

### Temozolomide treatment inhibits JAK-STAT pathway signaling

*PD-L1* expression is predominantly regulated by the JAK-STAT signaling pathway after stimulation with IFNγ. Alterations of this pathway were shown to be associated with resistance to immune therapy in melanoma [19]. We analyzed the JAK-STAT signaling by an expression target-panel of the JAK-STAT pathway including *Interferon-gamma-stimulated genes (ISGs)* such as *STAT1, STAT3 IRF1, IDO1, ITK, ICAM* and *CCL8*. In line with previously published studies [27], the mesenchymal cells showed an increased baseline-level of JAK/STAT signature genes, Fig. 2c. All three cell lines increased their pathway activation after IFNγ stimulation, Fig. 2c. Additional TMZ treatment altered the stimulating effect of IFNγ and lead to decreased expression-levels of the JAK-STAT target panel. This effect was only observed in the two primary cell lines (p < 0.001, Fig. 2c left panel) but not detectable in LN229 cells (Fig. 2c right panel). In order to validate our results on a protein level, a *PD-L1* Immunoblot was performed**,** Fig. 2d. Our findings showed a significant increase of *PD-L1* expression in the IFNγ stimulated groups as well as the previously described decrease of *PD-L1* expression after additional TMZ treatment, except in the LN229 cell line, Fig. 2d. An Immunofluorescence of the GSC168 cell line treatment groups was performed to visualize *PD-L1* expression and confirm an increase of *PD-L1* signaling after IFNγ administration (ctrl compared to IFNγ), Fig. 2e-f. In line with the western blot and expression analysis results, we showed a reduction of PD-L1 by combined IFNγ and TMZ treatment mainly caused by the decrease of STAT3 phosphorylation, Fig. 2f-g.

### Reduced JAK-STAT signaling in recurrent glioblastoma

In a next step, we aimed to analyze tumor specimens in order to detect the crosslink between *PD-L1* expression and the JAK-STAT pathway. Therefore, we examined tissue samples of de-novo and recurrent GBM of patients of the University Hospital of Freiburg. All patients had received the adjuvant standard-of-care Stupp-Protocol treatment after first diagnosis. Our JAK-STAT expression panel showed an increased intensity for JAK-STAT pathway activation in de-novo GBM tissue in comparison to recurrent samples after TMZ therapy, Fig. 2h. This downregulation consecutively resulted in significantly decreased *PD-L1* expression levels in recurrent samples. This effect was also confirmed in an immunoblot by PD-L1 and phospho-STAT3 protein levels, Fig. 2h. In addition, we analyzed 4 patients with exceptionally high PD-L1 levels in the de-novo tumors, selected from our tumor database, and their recurrent tumor tissue counterpart. Even though patients’ PD-L1 levels were individually distributed, we observed an overall decrease of PD-L1 and phospho- STAT3 protein levels after TMZ therapy, Fig. 2i.

In conclusion, all findings from qRT-PCR, Immunoblot and Immunofluorescence support our hypothesis: Temozolomide treatment can alter immune response by interfering with the intratumoral JAK/STAT pathway signaling. As *PD-L1* expression seems to be mostly controlled via the JAK/STAT pathway, the TMZ induced alterations may lead to a decreased capacity for *PD-L1* expression within the tumor cell.

## Discussion

Temozolomide (TMZ) is an alkylating chemotherapeutic agent [28] and established as adjuvant standard-of care treatment for GBM patients [9]. With the emergence of novel immune-checkpoint inhibitors for cancer therapy, an ongoing discussion addresses the role of TMZ as first-line treatment for GBM patients [10]. Especially MGMT non-methylated patients may not benefit from TMZ therapy, but will mainly experience adverse side effects such as myelo- and immunosuppression [29]. At the worst, these effects may even disrupt active antiglioma immune response and lead to poor results of immune checkpoint inhibitors like the PD-1/PD-L1 checkpoint inhibitor Nivolumab. In a previous study, we showed that an extended therapy with TMZ (above 6 adjuvant cycles) significantly lowers the PD-L1 expression in recurrent tumors independently from the MGMT promoter methylation [16]. We hypothesized that TMZ, in addition to its chemotherapeutic effects, might also induce changes in intracellular signaling cascades or other pathway activations, which leads to decreased PD-L1 signaling. Following this hypothesis, we aimed to investigate TMZ effects on *PD-L1* expression and its associated regulatory pathways in a glioma cell model. First, we used a publicly available database and analyzed differences between de-novo and recurrent GBM specimens. The analysis revealed a relatively constant expression of the typical subtype expression of proneural, mesenchymal and classical expression signatures Fig. 1b, bottom, which is in line with other reports [30]. The only outstandingly down-regulated pathways were inflammatory response and JAK/STAT activation, which has not been reported to far. In a next step, we aimed to confirm our findings in cell culture models from one commercial (LN229) and two primary, patient-derived cell lines. To respect any possible effects of GBM subgroup properties, both primary cell lines were classified beforehand by RNA-sequencing analysis [22]. We noticed, that a baseline *PD-L1* expression was only observable in the mesenchymal cell line. This is in line with previous reports that show a significant association between the mesenchymal subgroup and *PD-L1* expression [27]. The baseline *PD-L1* expression of the cell lines was most likely low due to the lack of inflammatory signaling within the cell culture model. Therefore, we used IFNγ stimulation to simulate a pro-inflammatory environment. In literature, this mechanism of IFNγ stimulation has previously been described to lead to an increased expression of *PD-L1* by activation of the JAK/STAT pathway [31, 32]. Among other pathways like the MAPK/ERK [33] and PI3K/AKT [17] pathway, several studies have identified the JAK/STAT pathway as the main pathway for *PD-L1* regulation [18, 31, 34]. TMZ was administered to the cells to mimic standard of care treatment and observe effects on the *PD-L1* expression and regulation. We showed that TMZ significantly reduces the *PD-L1* expression compared to the IFNγ stimulation. In line with previous reports that suggest an immune-modulating effect of TMZ [29], we hypothesized that TMZ interacts with PD-L1 upstream targets such as the JAK/STAT pathway.

We confirmed a reduction of the JAK/STAT pathway by combined TMZ and IFNγ. Different results were observed in the LN229 cell line, TMZ treatment did not induce a significant decrease of *PD-L1* levels, which could be explained by the character of the LN229 cell line. In line with many previous reports, commercial cell lines such as LN229 or U87 drift away from the “real” conditions in GBM tumors passage after passage and lose the properties of a naïve, patient-derived cell line [35]. Respectively, this may be a possible explanation for false positive or false negative results. As most studies on *PD-L1* are performed using commercial cell lines, this decrease of *PD-L1* expression after TMZ exposure has not been described before. Our findings may highlight the need for a more “personalized” approach to *PD-L1* analysis and the consecutive treatment choices. In a second step, we tried to verify the cell culture results by examining patient-derived brain tumor tissues of de-novo and recurrent samples. Even though all samples showed individual levels of *PD-L1* in the de-novo state, the expression was downregulated in all recurrent samples compared to the de-novo state. All patients had received standard-of-care treatment with TMZ after the first surgery. The extended qRT-PCR panel revealed a significant downregulation of the IFNγ- associated JAK/STAT pathway.

## Conclusions

In conclusion, this study reveals the crosslink between TMZ treatment and *PD-L1* downregulation in GBM cell lines and tumor tissues. TMZ seems to inhibit the JAK/STAT-pathway, which is one of the main pathways that controls *PD-L1* expression after stimulation by IFNγ. This hypothesis is supported by results from Zaretsky and colleagues, who showed a resistance to PD-L1 therapy in melanoma based on alterations in the pathways involved with Interferon gamma signaling [19]. As *PD-L1* has gained special interest in neurooncology as target for the immune-checkpoint inhibitor, a decrease in *PD-L1* expression might lead to disappointing treatment results. Especially the use of PD-1/PD-L1 checkpoint inhibitors in the recurrent state after TMZ treatment does not seem promising. Our study was limited by the small number of assessed cell lines (*n* = 3) and tumor tissue samples (*n* = 15). A closer analysis of the properties of commercial GBM- like cell lines compared to primary patient-derived cell lines might offer new and better insights to *PD-L1* regulation and quantification in vivo. As it is within the nature of a cell culture model, this study was only able to show TMZ effects in an artificial in vitro environment. An in-vivo brain tumor, influences on *PD-L1* expression are more complex, not yet fully understood and highly individual due to differences in immune system function, tumor biology and microenvironment. The exact mechanisms of *PD-L1* regulation and expression need to be further examined to understand and predict the benefits of immune-checkpoint inhibition for GBM patients.

## Abbreviations

GBM: Glioblastoma Multiforme
GSEA: Gene Set Enrichment Analysis
IFNγ: Interferon Gamma
JAK: The Janus kinase
MGMT: O-6-Methylguanine-DNA Methyltransferase
STAT: Signal transducer of activation
TMZ: Temozolomid
